# Quality of life returns from basic research

**DOI:** 10.1186/1478-4505-8-18

**Published:** 2010-06-07

**Authors:** Susan E Cozzens

**Affiliations:** 1 Technology Policy and Assessment Center, School of Public Policy, Georgia Institute of Technology, Atlanta GA 30332-0345 USA

## Abstract

**Background:**

Assessing the consequences of research is an increasingly important task in research and innovation policy. This paper takes a broader view of those consequences than the conventional economic approach, placing researchers and their activities in the centre of the assessment process and examining results for professional practice and general education as well as contributions to knowledge.

**Methods:**

The paper uses historical and documentary analysis to illustrate the approach, focusing on U.S. biomedicine over the past century. At aggregate level, the analysis attributes portions of the change in aggregate health indicators to research and research-based institutions, through several available types of logic: either through correlations between timing of institutional changes and changes in the indicators or through direct or indirect causal connections.

**Results:**

The analysis shows that while biomedical research has certainly contributed to improved health in the United States, other factors have also contributed. In some ways the institutional structure of science-based medicine has worked against creating benefits for some groups in U.S. society.

**Conclusions:**

The paper concludes with a call for more strategic attention to dimensions of impact other than knowledge outcomes and for participatory planning for research.

## Background

An earlier version of this article was originally presented in 1994 to a policy audience in Washington, DC. It appears here at the invitation of the editors because it raises issues that are still open in the assessment of biomedical research. The intervening years have seen the introduction of much stronger processes in the United States for strategic planning and accountability through evaluation, as the article advocates. Yet the broader dimensions of relationships between biomedical research and society outlined here have not been incorporated into those systems. The article's message is thus still pertinent; perhaps conditions are riper now for its acceptance. Updated information and additional references suggested by HARPS reviewers have been added in Table 1, to maintain the original flow of the argument.

**Table 1:** Selected Updated References

A	There is a large literature within medicine on quality of life measurement. For example, the Centers for Disease Control in the United States measure "health-related quality of life" in terms of healthy vs. unhealthy days, as reported on health surveys. "Unhealthy days are an estimate of the overall number of days during the previous 30 days when the respondent felt that either his or her physical or mental health was not good." (http://www.cdc.gov/hrqol/methods.htm, accessed January 1, 2010). CDC reports that "in recent years, several organizations have found these Healthy Days measures useful at the national level for (1) identifying health disparities, (2) tracking population trends, and (3) building broad coalitions around a measure of population health compatible with the World Health Organization's definition of health." (http://www.cdc.gov/hrqol/methods.htm, accessed January 1, 2010). In this article, I use the term quality of life goal in a broader sense, to indicate a family of public goals articulated for research. The health-related quality of life measure are one example of indicators that could be used to implement planning and evaluation for such goals. For example, they could be used at the aggregate assessment level described in a later section of the paper.
B	It would be interesting to compare the U.S. case with other national examples, such as the quite distinctive institutional developments in the United Kingdom. See Shergold, M and J Grant, **Freedom and need: The evolution of public strategy for biomedical and health research in England**, *Health Research Policy and Systems *2008, 6(1):2-12.

C	There is of course a substantial literature on the ways knowledge is co-produced with its social context. It is beyond the scope of this article to review that literature, but interested readers may wish to refer first to Nowotny, H, P Scott, and M Gibbons, *Re-Thinking Science: Knowledge and the Public in an Age of Uncertainty*. Cambridge: Polity Press, 2003.

D	This dimension corresponds to knowledge utilization, which again has its own signficiant literature. As a starting point into this literature, the reader may wish to consult the work of Carol Weiss, for example her 1980 article "Knowledge creep and decision accretion," in *Knowledge: Creation , Diffusion, Utilization *1: 381-404.

E	Science communication has its own large literature and a central journal, *Public Understanding of Science *http://pus.sagepub.com. The emphasis on teaching as a form of science communication taken in this article as it was first written calls attention to systemic structural aspects of the science communication system that get little attention in the science communication literature.

F	In the U.K., a whole new health research strategy was introduced in 2006, including institutional mechanisms designed to increase translational research and move research knowledge from "bench to bedside." For the strategy, see http://www.dh.gov.uk/en/Publicationsandstatistics/Publications/PublicationsPolicyAndGuidance/DH_4127127, accessed January 1, 2010. For a description of the work of one of the new institutions, see http://www.nihr.ac.uk/about/Pages/default.aspx, accessed January 1, 2010.

G	In the United States, the Washington Research Evaluation Network (WREN) played such a role; its efforts have been folded into an interagency group developing the Science of Science Policy (http://scienceofsciencepolicy.net, accessed January 1, 2010). There is an active branch of this community in the U.K. and the Netherlands, and the Swedish Research Council has helped to move the state of the art forward with two workshops (http://www.vr.se/forskningvistodjer/seminariedokumentation/medicin/workshopnewfrontiersinevaluationofimpactsofmedicalresearch.4.72e6b52e1211cd0bba880005128.html, accessed January 1, 2010).

Quality of life goals for science and technology have taken on new prominence in many industrialized nations in the post-Cold War period in the United States. National security has by no means disappeared as a reason for maintaining national technical capacity, nor have the benefits of research for innovation and economic competitiveness been discounted. But healthy, educated citizens and high-quality, high-paying jobs have moved up in the list of priorities. And equally important, new ways of reaching the older goals are being articulated. The United States is not only concerned to be among the world's leaders in science and engineering, but also to do so in a way that lives up to America's ideals of equal opportunity and utilizes all of its talents. Many nations are aiming not just for a changing world, nor just for a more technologically-intensive one, but also for a socially and environmentally sustainable one. Objectives have shifted from simply developing technology to doing so responsibly, and the goal of "wealth" has transformed into that of "prosperity."

My questions here concern how to achieve what we want in these added dimensions of scientific progress and technological change. What has basic research contributed to the quality of life as we define it today? How can we judge whether current efforts are succeeding by these criteria? How can we set goals and manage research to achieve the returns in quality of life that we want, for ourselves, for our children, and for our grandchildren?

## Methods

A crisp definition of quality of life goals eludes me in this article, as it has eluded and probably will elude public discussion. All true national goals are fuzzy and permit many definitions, because they represent broad consensus rather than scholarly precision. National security and economic competitiveness are goals of this sort, and quality of life need not be any clearer. Like other national goals, quality of life goals are translated through decision making processes into quality of life objectives. The objectives need to be proposed in context, debated publicly, and put into operation in relation to specific programs, rather than set by fiat in a paper such as this one. In addition, the term quality of life is likely to carry different meanings in different cultural contexts. (See Table 1, Row A for an additional context.)

In general, however, I use the term quality of life goals to apply to goals with the following characteristics:

• They apply to the way the full range of humans live, including their diverse work, family, home, and community lives.

• They refer to objectives with importance above and beyond their economic exchange value.

• They refer as much to the way we do science and use technology as to the content of science or performance characteristics of technology.

In short, they are goals that allow people to articulate what they need from scientists and engineers in order to live in a world of their own choosing.

Two kinds of goals that fit these criteria have emerged over the years in research policy discussions. I refer to them in this paper as *what *and *how *goals. Quality of life what-goals for research include health, education, and environmental quality. Quality of life how-goals include equity, democracy, and community. What-goals enter science policy discussion most often as program objectives. How-goals often enter as selection and evaluation criteria.

The article begins by describing the general approach I take to assessing progress toward quality of life goals. I then sketch how that assessment might look if we concentrated on one area of research, biomedicine, and looked back over two time perspectives in the United States:

• a century-long perspective, going back to the time when, in very approximate terms, science-based technology and medicine began to be institutionalized on a large scale in America;

• a half century perspective, approximately since the addition of government support to the institutional matrix for scientists in the United States.

Having looked to the past, I then turn toward the future, asking how we could use current research policy and management methods to maintain progress toward quality of life goals over the next twenty years, which seems about the right time frame for thinking about achieving quality of life results through shaping support for researchers today.

I use U.S. biomedicine as an extended example in my analysis (but see Table 1, Row B). Biomedicine has often been invoked as the exemplar of strategic research, that is, of fundamental science consciously organized to contribute in the long run to the solution of practical problems. Judging by the levels of direct public involvement in biomedical research policy, it is also clearly a high priority for a wide range of citizens in affluent countries. Every area of science, every public program that supports science, however, sits in a slightly different ecology of relationships with practice and education. In the final section, I discuss the applicability of the model I work with to other areas of science, and point toward needs for further research in this area.

### The Conceptual Framework

#### Bringing people back in

In policy discussions, research is often described abstractly: a national government supports "research"; "research" produces benefits for society. This form of language follows the conventions of scientific writing, which call on authors to remove themselves from their texts. But it distorts our assessment of the connections between research and quality of life by leaving the people who do science out of view. In this particular bit of imagination-stretching, I ask my readers to bring the people back in, for two reasons. First, when we see research as the concrete activity of specific individuals, whose jobs involve activities other than research and who also lead lives outside science, we bring both their institutions and their communities back into view as well. This step is important in assessing the full connections of research with the quality of life.

Second, we need to bring the people back in to re-orient our thinking about the purposes of government support for research. I treat research here as the advancement of knowledge in the sciences. But I treat the central goal of government support of fundamental research not as the production of knowledge per se, but rather the maintenance and renewal of national technical capacity in the form of researchers. By this, I do not mean that national governments should support researchers whether or not they are producing new knowledge; far from it. Instead, I call attention to the consequences for society that come from having researchers around, consequences that go beyond the production of knowledge.

One of the most important consequences of bringing the people back in to research policy is that it frees us from thinking in terms of targeting knowledge production. Instead of trying to plan what we need to know next to move toward a quality of life goal, we can plan where, in society or the economy, we want to develop technical capacity. That is, we ask, where do we want researchers to be, and who do we want them to be talking to? We can then leave the question of what specific questions they ask completely in their hands.

### A Three Dimensional Model

To be able to trace the connections between researchers and quality of life outcomes, I suggest a simplifying scheme [1]. We can trace the results researchers produce in three directions, which I will refer to as knowledge, practice, and education.

• The primary job of the researcher is to produce *knowledge *that contributes to a research front. The researcher's contributions then become part of a larger pool of knowledge. (See Table 1, Row C.)

• The long-term benefits we associate with research begin when someone draws on that pool of knowledge. That person might use the knowledge to improve *practice*, for example, in industry, medicine, or agriculture. What practitioners reads in the research literature can also change their concepts, frameworks, and world views, directly or indirectly. (See Table 1, Row D.)

• Or the person might draw on it in the context of *teaching *or *science communication*, to transmit the current best understanding of how the natural and technological worlds work to students and the general public. (See Table 1, Row E.)

It is precisely because the benefits of science come from the pool of knowledge rather than directly from an individual knowledge contribution that seeing the connections between basic research and the quality of life is so difficult when one thinks of research only in terms of knowledge outputs.

When one focuses on researchers themselves as the primary product of government support programs, however, the other routes through which benefits are accomplished spring into view. For example,

• the researcher may use his or her expanding expertise and familiarity with the knowledge pool directly in *practice*, by consulting (as engineers do often, with public and private organizations), by combining practice and research in the same job description (as medical researchers who are also active clinicians do), or by serving as an advisor to a governmental or non-governmental organization at national, state, or local levels.

• Some researchers, those who do graduate training, also contribute to practice by *training professionals*. When they leave their training in a research setting, professionals are up to date on the contents of the knowledge pool and equipped with the inclination and skills to dip into it later when they need it.

• In an analogous way, researchers on university campuses contribute to the utilization of the knowledge pool by providing up to date *undergraduate teaching*, and creating a life-long love of learning among college-educated people. Among Americans, for example, half go to college, and the other half are taught by people who went to college. The quality of undergraduate science education and the extent to which it creates a sense of competence with regard to technical matters thus has a very wide influence on quality of life in the United States.

I organize my discussion of the quality of life results of basic research along these three dimensions--knowledge, practice, and education.

### Approaches to assessing quality of life outcomes

Before turning to my example, biomedicine, I must ask the indulgence of my readers through one more set of rather abstract considerations, which they will want to follow in relation to the example. Quality of life outcomes of basic research, like its economic outcomes, can be assessed either at aggregate or at program level. The logic of the assessment is different at these two levels, and the way the two sets of analyses interact is important in building up a knowledge base on quality of life returns.

At the *aggregate level*, the assessment begins by identifying indicators of the relevant what-goals--in the biomedical case, for example, indicators of health. Given that the goals themselves are broad, multiple indicators will undoubtedly be available, and experts will not all agree on any single key indicator. But there is likely to be broad agreement, at least retrospectively, about the direction of progress implied in the indicators as a set.

The analysis then attributes portions of the change in the indicators to research, through several available types of logic. One can use the timing of the research contributions and change in the indicators, as, for example, in this analysis of the contribution of medical research to health outcomes:

The tide of infectious and nutritional diseases was rapidly receding when the laboratory scientist moved into action at the end of the past century.... In reality, the monstrous specter of infection had become but an enfeebled shadow of its former self by the time serums, vaccines, and drugs became available to combat microbes [2].

Alternatively, one can trace direct causal connections, for example, between the introduction of a vaccine and a subsequent dramatic drop in the incidence of a disease, or indirect causal connections, for example, through the facilitation of an approach or technique, or the incorporation of the knowledge into a technology.

A genre of research called "retrospective studies" has demonstrated the general usefulness of such explorations of the rich network of connections linking research with outcomes. Most early retrospective studies, however, started with specific advances in an area of practice and traced the events that led to them [3,4]. To judge quality of life outcomes, one must use a broader set of indicators, since some of the data may show lack of or the opposite of progress. Clearly, it is just as important to ask why some problems are getting worse and what research capacity we need to reverse those trends as it is to assess what research has contributed to past progress toward quality of life. It is also important to examine both how-goals and what-goals in such an analysis--that is, for example, not just whether biomedical research contributed ultimately to health, but whether it did so in a way that contributed to or detracted from equity, and contributed to or detracted from the empowerment of citizens.

At the *program level*, the analysis begins from the opposite end of the causal chains, in the activities of the researchers themselves. It tracks their immediate outputs--knowledge production, usually in the form of publications; direct contributions to practice and indirect ones through the training of professionals; and contributions to the educational stream through undergraduate teaching and general science communication activities. Using the set of institutional and social linkages that retrospective studies have identified, the analyst can then see where and how the researchers in the program are contributing, not in terms of direct production of outcomes, but by setting in motion the sorts of processes we expect to produce outcomes.

Individual programs have their own goals, of both the "what" and "how" varieties, and cannot be evaluated by whether they have moved the entire set of quality of life indicators in a particular direction. Government-level leadership, however, can compare the sum total of indicators in a particular quality of life area with the sum total of contributions and consequences that result from public programs and assess whether the portfolio of programs is connected richly enough to the institutions that produce what-outcomes and is operating in a manner that will produce how-outcomes. Thus aggregate retrospective studies combine with program assessments to give high-level decision makers the needed information base to manage for quality of life outcomes. I return to these activities in my discussion and conclusions.

## Results

### What has research contributed to quality of life?

We do not know as much as we could about the connections between researchers and the changing quality of life in America; but we do know some things. This section provides only a broad-brush view, based on commonly-accepted observations, to sketch in the categories of linkages discussed above in relation to the example of biomedicine. It does not provide an assessment itself, but rather points the way to one. I concentrate here on the what-goal of health, and the how-goals of equity and democracy.

### The century-long perspective

#### Researchers

In U.S. biomedicine, the century-long viewpoint takes us back to an era of a major reform in medical education in America [see [5-8]]. In the post Civil War years, doctors were a weak professional group in American society, competing with each other for a small market and with no special claim to authority over medical care. For admission to medical school, only a high school education was required. The medical curriculum followed no special order, and the graduation requirements were minimal. In 1871, Harvard led the way to raising standards, extending the academic year from four to nine months and the full course from two to three years. Other institutions found they could not afford not to follow suit. In 1890, the new Association of American Medical Colleges, which included the most progressive one third of medical schools, set minimum requirements of three years, six months a year, with laboratory work in histology, chemistry, and pathology.

In 1893, the Johns Hopkins University opened its medical school, embodying "a conception of medical education as a field of graduate study, rooted in basic science and hospital medicine, that was eventually to govern all institutions in the country. Scientific research and clinical instruction now moved to center stage" [[5], p. 115]. Hopkins recruited faculty nationally instead of locally; required two years of pre-clinical sciences and two on the wards; and created advanced residencies in specialized fields. We now talk of "partnerships" with regard to research: Hopkins invented them in biomedicine, establishing a hospital in connection with the medical school, joining science firmly to clinical hospital practice. Hopkins graduates exported this model around the country over the next decades.

Disparities grew between schools following this model and smaller, commercial or special-interest institutions, and reached a watershed early in the twentieth century. In 1910, a young Hopkins-connected educator, Abraham Flexner, was hired by the American Medical Association to study the quality of education at American medical colleges. His report recommended closing most of them: he would have kept only 31 out of 131. In the wake of the report, many did not survive, and those that did raised their standards. An influx of private philanthropic support reinforced this shift. Rockefeller's General Education Board channeled $91 million into medical schools over the next two decades, with seven institutions receiving over two thirds of the funds. Its staff actively encouraged medical education more closely linked to medical science than to medical practice. "These policies determined not so much which institutions would survive as which would dominate, how they would be run, and what ideals would prevail" [[5], p. 121].

#### Knowledge

Thus the seeds were sown for the post-War growth of government-sponsored biomedical research. Aside from the institutional base built through private philanthropy at a small group of research hospitals, only a few government-sponsored health-oriented research institutions existed before the Second World War. These included a small Laboratory of Hygiene that would grow up to be the National Institutes of Health.

Two important aspects of the pattern of biomedical research in the 20^th ^century appeared during this period. On the one hand, science within the medical school context was more likely to be linked to medical practice than science done outside; thus from the viewpoint of researchers themselves, the knowledge-practice link was forged in the Hopkins model. Assuming that research knowledge was to be useful to medical practice, the alternative was that researchers should be located outside medical institutions--clearly a second choice. On the other hand, under the science-based model as it actually developed, the technical capacity developed through research, embodied in the researchers themselves and those they trained, was highly concentrated in a few institutions. As a result, from the viewpoint of medical practitioners, a gap opened up between the new scientific medicine and general medical practice.

#### Practice

The gap took several forms. First, at the individual level, "academic and private physicians began to diverge and represent distinctive interests and values" [[5], p. 122]. Thus, while medical science could respond to clinical problems as they appeared in the context of the great teaching hospital, most doctors might find the research results distant and hard to assimilate into their practice. In addition,

the medical profession grew more uniform in its social composition. The high costs of medical education and more stringent requirements limited the entry of students from the lower and working classes. And deliberate policies of discrimination against Jews, women, and blacks promoted still greater social homogeneity. The opening of medicine to immigrants and women, which the competitive system of medical education allowed in the 1890s, was now reversed. [[5], p. 124]

Second, at the community level, the move to scientific medicine created great disparities. Flexner's recommendations to reduce the number of medical schools to 31 would have left twenty states without any medical schools. In the end, over 70 survived, and state legislatures stepped in to assure at least one institution in virtually every state. Even these institutions did not produce doctors for every community, however. "Before the Flexner report, there had been seven medical schools for blacks in the United States; only Howard and Meharry survived" the 1910 watershed. (For the impact of the Flexner report at Howard, see [9].) African Americans faced outright exclusion from internships and hospital privileges at most institutions. In 1930, only one out of every 3,000 black Americans was a doctor, and in the Deep South, the ratio was one in over 14,000. One can only imagine what the comparable numbers were for, for example, Asian Americans and American Indians. One might assume that European American doctors were providing adequate health care for African Americans as well, but segregation and racism must surely have intervened. (See the account in [10] as an example.) Doctors from communities that lost access to medical expertise in the wake of the Flexner report complained that while their local medical training might not be the equivalent of Harvard's or the University of Pennsylvania's, it was better than having no doctors at all in poor and rural communities. "Would you say," one wrote, "that such people should be denied physicians? Can the wealthy who are in a minority say to the poor majority, you shall not have a doctor?" [[5], p. 125] The implicit answer was, "Yes."

#### Education

Another consequence of the way U.S. biomedical research was established institutionally was its isolation from the general educational stream. Biomedical researchers were medical school faculty, and did not generally teach undergraduates, even at Harvard, Johns Hopkins, or any of the other universities where they were located. What biomedical researchers were learning about basic biology, then, had to be diffused through the general pool of biological knowledge before it could reach college students, and through them the school curriculum. The routes for diffusion of research knowledge to general medical practitioners were strong in comparison. Thus the institutional location of biomedical research contributed to a growing gap in expertise between medical practitioners and their patients.

#### Outcomes

From the advent of scientific medicine to the Second World War, the average health of Americans certainly improved. But medicine itself, and scientific medicine in particular, is generally credited with only a small part of this improvement. A working group of the Carnegie Commission on Science, Technology, and Government gives considerable credit to factors other than research:

The health of the American people, as judged by life expectancy, has been improving since the turn of the century. Initial improvements in longevity primarily reflected diminished mortality from infections and were largely attributable to improvements in sanitation and nutrition and to the development of effective vaccines. Sulfonamides, penicillin, and other antibiotics contributed to a further decrease in death rates [11].

In short, specific preventive and therapeutic measures developed during this period probably only accelerated a decline in mortality rates that was already underway.

To focus only on such aggregate assessment of the products of biomedical science, however, misses half of the challenge of examining quality of life issues. What matters is not only what we have done, but how we have done it. To complete this analysis, we would have to ask about the differential distribution of health outcomes among the American people. We would have to take into account the continuing exclusion of many population groups from the medical profession generally and from biomedical research careers specifically in the first half of the twentieth century. And we would also have to take into account the relationship of patients and their families to doctors and the medical system generally. I return to this topic in the next section.

### The half century perspective

#### Knowledge

Research within the medical context received a tremendous boost in the post-War period, and continues to dominate the profile of government-sponsored basic research. The story is quite well known. Following stunning contributions to the war effort, researchers gained dominance in the Public Health Service in the late 1940s. Two wealthy women, Mary Lasker and Florence Mahoney, raised the public profile of biomedical research and stimulated a coalition among researchers, members of Congress, and segments of the mobilized public, which resulted in a phenomenal growth rate in funding in the 1950s. The new biomedical coalition adopted a strategy of seeking funds under the rubric of specific diseases, and the now-plural National Institutes of Health subdivided rapidly on this basis. Researchers retained a remarkable degree of control over research, through institutional mechanisms such as peer review (see discusion in [5], drawing on [12,13], and [14].)

In terms of the content of knowledge itself, by far the most prominent trend has been the move toward the molecular level of analysis. Within the framework of disease-oriented institutes, biomedical researchers are now more tied to each other in their exploration of the fundamental dynamics of genetic expression. Moving to this level of analysis has also reunited biomedical knowledge with the rest of biology more powerfully than before.

#### Researchers

The enormous quantitative increase in the amount of biomedical research activity has brought with it a broader, more complex institutional base of researchers. Three quarters of NIH's current extramural research is done in higher educational institutions; just over half of the support goes to medical schools alone [15]. Outside the medical schools, large numbers of applications come from biology, chemistry, and biochemistry departments, but two thirds are spread across the rest of the university. About 20 percent of extramural funding goes to non-profit research institutes, independent hospitals, and other non-profit institutions. Geographically, while the funds are by no means distributed evenly on a per capita basis, every state gets something: even Wyoming, ranked last, had over $7 million in NIH funds in 2007 [16]. About one in four NIH applications has traditionally been from a person with a medical degree [17]. Industry has also built its technical capacity by hiring biomedical researchers, a trend that was only beginning in the 1930s and did not become a major activity until after the war. Pharmaceutical firms have significant in-house research efforts, and the medical device industry is also quite research-intensive, using researchers with many kinds of expertise including biomedical.

Minority communities, however, continue to lack access to this technical capacity. Biomedical research is still largely a white domain in America, more white than medical practice itself. In the 1990s, an advisory body of minority researchers and practitioners chose building minority biomedical technical capacity as the keystone of the Minority Health Initiative at the National Institutes of Health. A counterpart group considering women's health issues had focused on the shorter-term strategy of addressing the knowledge base with regard to specific female health problems like menopause and breast cancer; only in later rounds of resource allocation did the question of training programs for female researchers arise. But the minority group allocated over half of the available resources from the beginning to training programs, thus affirming the perceived importance of the equitable distribution of technical capacity among Americans [18,19].

#### Practice

The connections between present-day biomedical research and various kinds of practice are clearly rich. Biomedical researchers themselves consult, own biotechnology and drug firms, and are practicing physicians, among other roles. In medical schools, they train new generations of doctors and provide continuing education for doctors already in practice. Through graduate training for researchers who end up in industry and continuing collaborative relations with industrial researchers, they build the technical capacity of pharmaceutical and other firms, maintain personal connections between industrial and academic research, and increase the likelihood that industrial researchers will have the knowledge and inclination to draw on the pool of biomedical knowledge as they need to in their work. As we saw in the earlier period, from the viewpoint of researchers, institutional locations create a powerful network keeping them aware of practical problems and providing channels for their research results to be incorporated into practice.

From the wider viewpoint of medical practice, however, the network may not appear so effective. Certainly a large proportion of physicians are learning the basic sciences at a high level in their medical curriculum, and are therefore prepared as well as possible for the struggle to keep up with research results that appear after they are in practice. As the pool of biomedical knowledge spreads to become a lake and then an ocean, however, the challenge of continuing education for physicians looms large. The physician's most common information on research results may be the media and drug salesmen. As doctors move through their three decades or so of practice, they inevitably find themselves further and further from the research front, and incorporate new therapeutic approaches with more difficulty and less understanding into their work.

Some of the most controversial outcomes of the growth of biomedical research are mediated through the training of medical practitioners and the symbiotic relationship between science-based medicine and medical technology. There is a growing body of anthropological evidence examining the relationship between patients and the medical system as a factor shaping a personal sense of autonomy and empowerment. In this analysis, the growing scientific knowledge of medical practitioners can increase a sense of powerlessness in patients. This sense is exacerbated by medical technology, which transports one's experience of one's own body outside the body, onto imaging screens and into numerical test results. The women's health movement has been particularly vocal in drawing attention to the disempowerment that can result from standard doctor/patient or patient/hospital interactions (see, for example, [20-22]). The point applies with even more force to poor communities, whether rural or urban. Given the number of interactions Americans have over a lifetime with medical care providers, any such sense of disempowerment can accumulate to the point of leading to a larger sense of alienation from authority. The situation is thus of no inconsiderable consequence for general levels of democratic participation.

#### Education

One clear benefit from the vast growth of biomedical knowledge and the push to the molecular level is the increased probability of integrating biomedical results with those of the rest of biology. As mentioned, biomedical research is no longer confined to the medical schools within the university. Biology and chemistry teachers, who regularly teach undergraduates, are also contributing to the biomedical effort. Thus it is not surprising that the basic results of biomedical research are making their way into college textbooks, and thus on to school level science training. The limitations on this diffusion route come largely from the problems of the educational system itself. The gap between what biomedical researchers know about the human body and what high school students in the nation's weakest schools know is undoubtedly widening. Likewise, other parts of the public are differentially exposed to new concepts in medicine. Most of modern genetics, for example, has been discovered in the time since the President's generation took high school biology. Public broadcasting documentaries, science sections of newspapers, and science museums combined do not reach more than a small segment of that generation with the simplest of knowledge of the major themes of contemporary biomedical research. Physicians and other health care providers can end up being the major science educators, but in relation to specific conditions and at times of stress. Thus the physician/patient relationship takes on yet another significant role in shaping the character of life with science for Americans.

#### Outcomes

An overall assessment of the outcomes of post-War biomedical research is of course a matter for debate and discussion. The high cost of health care is an issue: per capita health care spending in the United States has increased at a faster rate than per capita income for a number of decades [23]. And whether the gains in health were related to those costs, or to the investment in biomedical research, is controversial. Again I quote from the Carnegie Commission report as a sympathetic summary:

In recent years, increases in life expectancy have resulted primarily from reductions in cardiovascular death rates from stroke and coronary artery disease. These improvements reflect control of hypertension, a decrease in the prevalence of smoking, decreases in the intake of fats and cholesterol, better weight control, and healthier lifestyles. There have also been substantial reductions in death rates from certain types of cancer, owing to improvements in surgery, radiation, and chemotherapy. Unfortunately, increases in lung cancer due to smoking have approximately canceled out the successes with other forms of cancer. [[11], p. 42]

The aggregate numbers again mask differences in outcomes for different groups in the population. Between 1980 and 1990, the overall life expectancy at birth for Americans increased from 73.7 to 75.4 years. But life expectancy for the white population increased by 1.7 years and in the black population by 1.0 year, thereby widening the gap between the two. In 1990, the infant mortality rate for Americans as a group was 9.2 deaths per 1,000 live births, but it was 7.6 for white mothers and 18 for black mothers--and again the gap had widened between 1980 and 1990 [[24], p.1].

## Discussion

### The next twenty years

It is worth pausing at this point to examine how my analysis has proceeded. I have tried to raise the issue of the relationship of biomedical research to quality of life through several strategies. By using the perspectives of century and half century, I have forced us to look at the issue in terms of long-term relationships built up through institutional structures and patterns of interaction shaped by education, rather than just in terms of research results and their incorporation into medical practice. By focusing on researchers instead of research knowledge, I have reinforced the focus on institutions and the distribution of technical capacity among Americans and American communities. By using health statistics, I have forced us on the one hand to think about the larger set of practices through which the benefits of biomedical research must reach the public, and in particular about the distributive aspects of the medical care system. On the other hand, by talking about health, and not just health research, we have been forced to put the specific agendas and accomplishments of particular research communities into a broader perspective of life in America. The resulting discussion bears certain resemblance to now-familiar aggregate assessment of the contribution of basic research to the economy.

As with aggregate economic assessment, however, such broad-brush assessment of quality of life issues provides little direction to researchers, research managers, or policy makers in how to achieve quality of life goals. The question becomes pressing: How do we get from where we are now to where we want to be? I argue in this section that the conventional tools of research policy and management--goal-setting and program evaluation and planning--are appropriate to this task and that there has been progress in putting them to work.

Quality of life goals do put at least two new new requirements on research policy, however. The first is to think in terms of long term institutional and community development - something akin to the 100-year view in the analysis presented here. The second is the inclusion of the public--or to be more precise, publics--in all these processes. Just as we brought people back in to the concept of what government support for research is about, we must bring people back in to research management. Researchers themselves are not the experts on quality of life outcomes; they are partners in producing them. All the partners need to be involved in running the firm.

### Goal setting

In the years preceding the first version of this article, the variety and insistence of calls to set goals for research in the United States had been striking. The Carnegie Commission report, already quoted, stressed the need to set long-term goals, rather than squander technical resources on short-term objectives with no vision of the future in mind.

If this emphasis continues, the problems we have encountered in recent years, such as erosion of the nation's industrial competitiveness and the difficulties of meeting increasingly challenging standards of environmental quality, could overwhelm promising opportunities for progress. However, we believe there is an alternative. The United States could base its S&T policies more firmly on long-range considerations and link these policies to societal goals through more comprehensive assessment of opportunities, costs, and benefits. [[11], p. 11]

The report gave a long list of examples of major societal goals to which science and technology contribute, including personal and public health and safety, creation and maintenance of civic culture, and environmental quality and protection. It proposed a National Forum on Science and Technology Goals to facilitate "a balanced and effective interaction ... between the scientific and engineering communities and those representing a broad range of other societal interests." [[11], p. 13]

The call for goals had also been voiced within government. An influential senator had called for 60 percent of the National Science Foundation's budget to be devoted to strategic research, in areas such as high performance computing, biotechnology, materials science, and manufacturing [25], and the White House had taken up the challenge in its guidance for the budget process [26]. For biomedical research, a goals document of approximately the kind envisioned in the Carnegie Commission report existed: Healthy People 2000: National Health Promotion and Disease Prevention Objectives. "After extensive public review and comment, involving more than 10,000 people, the objectives were revised and refined to produce" the final report in 1990 [[27], title page]. The process of producing the report would seem to be a model for the sort of National Forum the Carnegie Commission had recommended.

Still, Healthy People 2000 illustrated the lack of connection between research program planning and long-term national goal setting. The Carnegie Commission noted the problem:

There is a mismatch between the long-term societal goals necessary for our society's well-being in the 21st century and many of the present scientific goals of research. The implications for biomedical research of a new social goal of cost-effective and equitable health care delivery to the entire U.S. population have not yet been carefully analyzed. [[11], p. 43]

The first version of an NIH strategic plan, announced to the press in the summer of 1992 but never released, seemed not to have taken Healthy People and its national goals into account.

Since the mid-1990s, strategic planning with stakeholder consultation has become a requirement for all U.S. federal government agencies, under the Government Performance and Results Act and later versions of results-oriented management. Healthy People 2010 was constructed in this environment, again through a broad participatory process [28]. Under Congressional mandate, the National Institutes of Health now produce strategic plans every three years and its budget reflects the goals and objectives of the plan. The NIH budget for 2008, for example, reported briefly its connection to Healthy People 2010 along with the strategic plan of its parent department, Health and Human Services, referring the reader to its performance goals for details [29]. But one can see little direct connection between the very general strategic priorities of NIH ("support new investigators," a bridge award, and a common fund for new initiatives [[30], p, 4]) and those of Healthy People 2010 ("increase quality and years of healthy life" and "eliminate health disparities." [28]) The intersections begin to appear among NIH's "strategic research objectives" and other performance goals. But among the 70 specific objectives (chosen as illustrative, not comprehensive), only two refer directly to the Healthy People document. A few more are aimed at "health disparities" or "underserved groups." But still it appears that even a government-wide effort asking agencies to articulate strategic goals has not produced much obvious alignment between the national health plan and the biomedical research plan for the United States (see Table 1, Row F for a related U.K. example).

### Program planning and evaluation

The question of how to get from here to our goals is actually answered in the process of program planning. In theory, this process translates broad government goals and objectives into specific program activities and budget requests. In practice, over the past decade, this process should have been engaging agency program planning much more powerfully with national goal setting, because of the new planning and budgeting processes introduced in the mid-1990s.

The language of planning tends to raise fears among scientists that policy makers are asking them to plan research. When we think of the goal of a research program as putting active researchers in certain kinds of contexts, however, the reason for the fears disappears. When we bring people back in, program planning for basic research programs no longer involves controlling the contents of research projects, but instead pays attention to where researchers are working, who they talk to, what communities they empower, and what else they do besides research.

Exactly what these criteria entail will vary from program to program. NSF's Engineering Research Centers and Science and Technology Centers serve as an example of how this approach can be used; the new U.K. Biomedical Research Centres are another [31]. The NSF Centers are not told in detail what to study, but they are required to show that they are embedded in a set of partnerships that is likely to move their research results into practice, and they are required to make a commitment to undergraduate education as well as graduate. When these broader functions of research become the focus, it is important to think in terms of aggregates of researchers rather than individual ones; no individual researcher needs to perform equally on all three dimensions of evaluation, but rather a set of researchers should do so. Thus it is the sum of centre-affiliated researchers that maintain the connections in three directions: knowledge, practice, and education. Likewise, any government research program can be seen as a sum of researchers, who together contribute to the knowledge base, maintain connections to practice, and enhance education.

The difference between a centre and a program, in this comparison, is that the centre is required to have a strategic planning process that directs research toward topics that the specific clients of the centre find important. Researchers and clients together participate in that process. A program consisting of a portfolio of individual grants (from individuals or teams) does not need to determine centrally what topics the investigators study. But it does need processes in which researchers and their partners together discuss how the total research ongoing in the program relates to its primary contexts of use, so that researchers themselves remain aware of these contexts and can take them into consideration in their own choice of research topics. The process of program planning provides one such opportunity, but the investigators themselves are seldom involved in that process. Program evaluation processes, however, which usually involve extensive interaction with program participants, offer a better setting for this negotiation [32].

Program evaluation for a program aimed at technical capacity asks, not "Have the researchers in this program answered a specific question?" nor "Have they achieved some particular result?" but

• Are they doing excellent research?

• What are they learning from it, and who else is learning it?

• Who draws on the pool of knowledge these researchers contribute to?

• Are these researchers talking to the people who use that pool of knowledge, to remain aware of the long-term practical problems it relates to?

• Are these researchers empowering citizens and consumers, directly or indirectly, with the knowledge they produce and how they produce it?

The information gathered in the evaluation can be used to shape the program, for example, by shedding light on the mix of resources investigators need to do excellent research and facilitate its use, or by identifying new partners or ways of interacting with partners that can increase the program's effectiveness. The evaluation process itself raises the awareness of both researchers and partners to the way the program actually works to contribute to quality of life goals, that is, how it sets processes in motion that end up in those results. In an untargeted program, the process of evaluation thus serves some of the same functions that the strategic planning process serves for a centralized research unit, but does so in a way that leaves choice of research topics and judgment of technical excellence entirely in the hands of researchers.

To achieve quality of life goals through such programmes, however, the key is who is involved in program evaluation and planning. To see the whole set of linkages through which the program is having its impact, evaluation panels need only a minority of researchers, but a majority that includes a wide range of people with other kinds of knowledge. One important set of such people is usually called "next-stage users" of research. These are people with knowledge of practice applications (like physicians, nurses, and counselors in biomedical research). The other important set of participants is end-users, that is, those who have experienced and thoughtfully considered the ultimate results of the researchers' work. For biomedicine, all members of the public are potentially end-users in this sense. Just as with the selection of researchers for evaluation committees, the challenge is to find participants with broad enough perspective and relevant experience to cover the range the committee needs. Finding such people is worth the effort, however. Unless the processes of program evaluation and planning include quite a wide range of such partners, they will not be able to judge quality of life outcomes effectively.

At the highest level, NIH has increased public involvement in shaping its programs. The NIH Director established the NIH Council of Public Representatives in 1999 [33]. The group includes medical practitioners and health advocates from a variety of disease and population groups. The Council issued a report in 2004 on enhancing public involvement in priority setting [34], urging the biomedical research community to "active listening" to those it intends to serve. The Council sets a new standard for NIH in public involvement.

**Figure 1:**
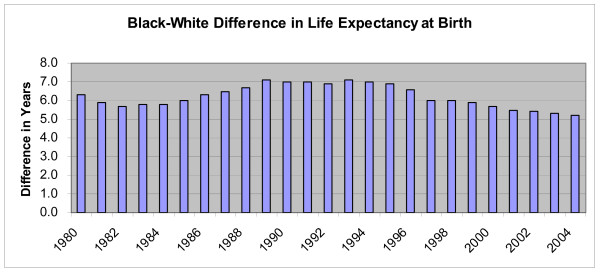
**Black-White Difference in Life Expectancy at Birth**. Source: http://www.cdc.gov/nchs/data/hus/hus07.pdf#027, accessed 6 July 2008.

The earlier analysis reported health outcomes for the decades examined. It would be naïve to trace any of the small changes we have seen in biomedical planning and evaluation directly to health outcomes in the United States. Nonetheless, let us end with at least one sign of movement in the right direction. Although the gap in life expectancy between white and black Americans remained unacceptably large, at least in the mid-2000s, the trend was in the right direction [Figure 1].

## Conclusions

The last section began to address the question of whether the framework I have been developing applies outside biomedicine. Parts of it, in particular the link between researchers and practice goals, are well developed in U.S. military R&D. Next-stage users are involved in many planning and evaluation processes for research there and in industry-oriented programs. End-stage users are involved in a few, including some military R&D assessment processes and NIH's councils. In disciplinary science programs in the United States, a different mix of the three dimensions is appropriate, with more emphasis on educational responsibilities, more diffuse relationships to practice, and more involvement of users within the science community, drawn both from the discipline supported by the program and from other disciplines that depend on its knowledge pool. Any government program of research can be seen profitably within the framework, however, as long as the appropriate weight is put on each of the three dimensions.

Two related bodies of knowledge and experience need to be built up extensively, to put this framework into practice effectively. First, we need to know much more about the activities and institutions that link basic research to quality of life outcomes. These are identifiable in specific cases, but there is no general body of knowledge, equivalent to the body of knowledge about industrial innovation processes, to provide general concepts in this area. Without general concepts, every retrospective study and evaluation process starts *de novo *to identify these links, and the overall effort required is much greater. A small community of researchers devoted to understanding these links is developing, through workshops and research projects, to play a role for quality of life outcomes that parallels the role of economists of R&D in relation to industrial innovation (see Table 1, Row G).

Second, we need to build up experience with locating appropriate public members of evaluation and planning panels, and utilizing their insights effectively. NIH has traditionally looked to organized patient and patient-family groups as members of its councils, as well as non-science professionals who bring different perspectives to the process. The new Council of Public Representatives strengthens this system. The U.K. has been working actively on public involvement for decades, through an effort now called INVOLVE [35]. These are positive steps, and can provide the beginnings of creative thought about broader representation. 		Once representatives of various public are on these panels, however, chairs and executive secretaries can easily be at a loss as to how to give them an effective voice. In the absence of a set of guidelines for effective public-researcher interaction in research decision making processes, such committees are open to unconscious domination by the research community, a process that defeats the purpose of public representation. If we are serious about achieving quality of life returns from basic research, we cannot afford to lose the benefit of the expertise of public members of these groups. We should therefore make concerted attempts to collate the experience to date with mixed committees of this sort, and build a base of research knowledge that can provide practical guidance to chairs and executive secretaries of future committees.

There is thus a great deal of work to be done to use the framework I have discussed in this paper to increase the effectiveness of programs with regard to quality of life goals. The challenges involve both of the ways I have discussed here of bringing people back in.

• Putting the researcher back at the center of research evaluation and planning concepts. We have focused too long and too exclusively on the knowledge products of research. Thinking about researchers as multi-dimensional contributors will take some imagination, and then some effort in translating that understanding into appropriate program structures and resources.

• Putting the public back in evaluation and planning processes. Public involvement in research management has been seen for too long as a threat to autonomy and a form of political control. In the framework I have outlined, the opposite is true. Public involvement in program evaluation and planning may be the only route, under current circumstances, to leaving researchers in control of their research topics and processes. Furthermore, it is a necessary condition for using technical capacity to contribute to quality of life goals, since researchers themselves have part of the expertise, but not all the expertise, needed to identify the complex links between research and these outcomes.

In short, I call for a quiet revolution in science policy, a transformation within existing structures and processes that will maintain, utilize, and expand the important strengths of current basic research systems.

## Competing interests

The author declares that they have no competing interests.

## Authors' contributions

Single author.
